# JNK and NADPH Oxidase Involved in Fluoride-Induced Oxidative Stress in BV-2 Microglia Cells

**DOI:** 10.1155/2013/895975

**Published:** 2013-08-29

**Authors:** Ling Yan, Shengnan Liu, Chen Wang, Fei Wang, Yingli Song, Nan Yan, Shuhua Xi, Ziyou Liu, Guifan Sun

**Affiliations:** Department of Occupational and Environmental Health, Liaoning Province Key Lab of Arsenic Biological Effect and Poisoning, School of Public Health, China Medical University, District of Heping, No. 92 North Er Road, Shenyang, 110001, China

## Abstract

Excessive fluoride may cause central nervous system (CNS) dysfunction, and oxidative stress is a recognized mode of action of fluoride toxicity. In CNS, activated microglial cells can release more reactive oxygen species (ROS), and NADPH oxidase (NOX) is the major enzyme for the production of extracellular superoxide in microglia. ROS have been characterized as an important secondary messenger and modulator for various mammalian intracellular signaling pathways, including the MAPK pathways. In this study we examined ROS production and TNF-**α**, IL-1**β** inflammatory cytokines releasing, and the expression of MAPKs in BV-2 microglia cells treated with fluoride. We found that fluoride increased JNK phosphorylation level of BV-2 cells and pretreatment with JNK inhibitor SP600125 markedly reduced the levels of intracellular O_2_^·−^ and NO. NOX inhibitor apocynin and iNOS inhibitor SMT dramatically decreased NaF-induced ROS and NO generations, respectively. Antioxidant melatonin (MEL) resulted in a reduction in JNK phosphorylation in fluoride-stimulated BV-2 microglia. The results confirmed that NOX and iNOS played an important role in fluoride inducing oxidative stress and NO production and JNK took part in the oxidative stress induced by fluoride and meanwhile also could be activated by ROS in fluoride-treated BV-2 cells.

## 1. Introduction

Fluorosis a progressive degenerative disorder resulted from excessive intake of fluoride (F) either by natural or by anthropogenic sources and most commonly through drinking-water. Endemic fluorosis is prevalent in many parts of the world and often seriously impairs the health of human or animals. Fluorosis causes damage not only to skeletal tissue and teeth but also to soft tissues such as brain [1]. Several studies indicated that excessive exposure to fluoride may be associated with central nervous system dysfunction. Animal studies have shown that chronic or subchronic fluoride exposure may lead to changes in behavior and neurodegeneration [2–4]. Intelligence Quotient (IQ) in children with exposure to high fluoride levels in drinking water was reduced [5–7]. Although the toxic effects of fluoride on the central nervous system were found, the specific mechanisms remained unknown.

Oxidative stress is a recognized mode of action of fluoride, and some studies have reported that fluoride can induce oxidative damage in brain tissue [8]. The central nervous system (CNS) is especially sensitive to free radical oxidative damage as it contains more easily oxidizable fatty acids [9, 10]. Reactive oxygen species (ROS) and free radicals played a key role in the pathogenesis of many diseases, including neurodegenerative processes [11, 12]. Oxidative stress is a prominent feature of many neurodegenerative disorders. Our previous study has found that fluoride can activate microglia and release ROS and reactive nitrogen species (RNS) [13]. 

Microglial cells are the resident macrophages of the brain and play an important role in immune responses in the brain. In the normal CNS, microglia display a quiescent phenotype, characterized by low expression of the cell surface molecules like CD14 and CD45, absent phagocytic activity [14, 15]. They are activated in response to brain injury from inflammation, damage, or disease and accompany the release of proinflammatory factors such as tumor necrosis factor (TNF-*α*) and interleukin (IL)-1*β*, as well as RNS, ROS, which also can exacerbate neuronal injury [16, 17]. ROS has been characterized as an important secondary messenger and modulator for various mammalian intracellular signaling pathways, including the MAPK pathways, namely, extracellular signal-regulated kinase (ERK), c-Jun N-terminal kinase (JNK), and p38 MAPK. In neuronal cells, ERK1/2 is primarily activated by growth factors and is involved in cellular proliferation, differentiation, and development, whereas JNK and p38 signaling cascades are preferentially activated by environmental stress and inflammatory cytokines and have been shown to promote neuronal cell death [18]. Several studies have shown that LPS induces the expression of iNOS and NO production via a mechanism mediated by the JNK pathway in microglia BV-2 cells [19, 20]. MAPK family which are responsive to stress stimuli are also involved in the generation of ROS and free radicals [21]. Some literatures reported that fluoride can induce the phosphorylation of all members of MAPK family in human pulmonary epithelial cells [22, 23]. Therefore, MAPK signal transduction pathway has become an important mechanism of fluoride toxicity and provided investigation clues for the pathogenesis of fluorosis. 

NADPH oxidase (NOX) is a multicomponent enzyme complex with the capacity to produce the highly reactive free radical superoxide. NOX is the major enzyme for the production of extracellular superoxide in immune cells and is highly expressed in microglia. In the present study, we examined ROS production and the expression of MAPKs in BV-2 microglia cells treated with fluoride and found that JNK and NOX were involved in the production of ROS in BV-2 microglia cells cotreated with fluoride and inhibitors of JNK and NOX. The results elucidated the possible molecular mechanisms of fluoride-induced ROS generation and provided experimental data for understanding the mechanism of CNS damage caused by fluoride.

## 2. Materials and Methods

### 2.1. Chemicals and Reagents

Sodium fluoride (NaF, molecular weight 41.99) was procured from Sigma Chemical (St. Louis, MO, USA). PBS, medium, antibiotic, and antimycotic solutions (10,000 U/mL penicillin, 10 mg/mL streptomycin, and 25 mg/mL amphotericin B) were purchased from GIBCO (Grand Island, NY, USA). Anti-ERK, anti-p38, anti-JNK, and anti-phospho-JNK antibodies were obtained from Cell Signaling Technology (Beverly, MA, USA). JNK inhibitor SP600125 was purchased from Calbiochem (La Jolla, CA, USA) and NOX inhibitor apocynin (API) was purchased from ChromaDex (Santa Ana, CA, USA). iNOS inhibitor SMT (S-methylisothiourea sulfate) was obtained from Beyotime Institute of Biotechnology (Shenzhen, China). 4′,6-Diamidino-2-phenylindole (DAPI) and all other analytical laboratory chemicals and reagents were obtained from Sigma, Invitrogen (Carlsbad, CA, USA), Hyclone (Logan, UT, USA).

### 2.2. Cell Culture and Treatment

 The immortalized murine microglia cell line, BV-2, was provided by cell culture center, School of Basic Medicine, Peking Union Medical College. The BV-2 cells were maintained in Dulbecco's Modified Eagles Medium (DMEM) that contained 10% fetal bovine serum and antibiotics at 37°C in a 5% CO_2_ humified incubator. Prior to each experiment, cells were plated in 24-well plates at a density of 1 × 10^5^ cells for culture supernatant tests or in 6-well plates at a density of 2 × 10^5^ cells for protein extraction. Exponentially growing BV-2 cells were pretreated with 2 mM melatonin (MEL) or 100 *μ*M NOX inhibitor API or 300 *μ*M iNOS inhibitor SMT or 50 *μ*M of JNK inhibitor SP600125 for 2 h and followed by treatment with 1–100 mg/L NaF for the indicated amount of time.

### 2.3. MTT Cell Viability Assay

 Viability and growth patterns of BV-2 cells based on mitochondrial enzyme functions in 96-well plates were determined under NaF-treated for 6, 12, 24, 48, and 72 h by 3-[4,5-dimethylthiazol-2-yl]-2,5-diphenyltetrazolium bromide (MTT) conversion to formazan. Briefly, 100 *μ*L of MTT solution (0.5 mg/mL in the medium) was added to each well, and the plates were incubated at 37°C for additional 4 h. Afterwards, the medium containing MTT was removed and the crystals were dissolved in 150 *μ*L of 100% dimethyl sulfoxide (DMSO). The cell viability was determined by measuring the optical density (OD) at 570 nm using a quantified microplate reader (Multiscan Ascent, Labsystem, Finland). All determinations were confirmed by replication in at least three independent experiments. 

### 2.4. Immunocytochemistry

Following NaF treatment, cultured microglia were fixed with 4% paraformaldehyde and incubated with 1 : 100 diluted primary antibody, rabbit anti-mouse Iba-1 (ionized calcium-binding adaptor molecule 1, microglia marker) for 1 h at 37°C, and secondary antibody Tritc-conjugated goat anti-rabbit IgG was added for 40 min at 37°C. Then nucleus was dyed with DAPI for 10 min. Staining of Iba-1was examined by fluorescence microscopy. 

### 2.5. Analysis for Intracellular ROS and O_2_^·−^

To measure the intracellular generation of ROS, the fluorescent marker DCFH-DA was used. Briefly, BV-2 cells in 24-well culture plates, preincubated with NOX inhibitor API for 2 h, and then treated with 50 mg/L NaF for 24 h, were rinsed and resuspended in serum-free medium containing 10 *μ*M DCFH-DA. After a further 30 min of incubations at 37°C, cells were rinsed twice with ice-cold PBS and harvested by trypsin. DCF fluorescence intensity was measured via a FACScan flow cytometer (BECTON DICKINSON, USA) with excitation wave length at 488 nm and emission wave length at 525 nm for ROS generation. The fluorescence intensity parallels to the amount of ROS formed. Intracellular O_2_^·−^ was detected by measuring dihydroethidium (DHE) fluorescence. BV-2 cells were harvested after being preincubated with NOX inhibitor API or 50 *μ*M JNK inhibitor SP600125 for 2 h and then treated with various concentrations of NaF for 24 h, washed with serum-free culture medium, and incubated with 5 *μ*M DHE at 37°C for 30 min. Then the cells were harvested, washed, and resuspended and were measured via a FACScan flow cytometer (BECTON DICKINSON, USA) with excitation wave length at 300 nm and emission wave length at 610 nm for O_2_^·−^ generation. 

### 2.6. Measurement of Intracellular NO Formation

The production of NO was determined by measuring 3-amino,4-aminomethyl-2′,7′-difluorescein, diacetate (DAF-FM diacetate) fluorescence. BV-2 cells were harvested after being preincubated with 300 *μ*M iNOS inhibitor SMT or 50 *μ*M of JNK inhibitor SP600125 for 2 h and then treated with various concentrations of NaF for 24 h, rinsed, and resuspended in serum-free medium containing 5 *μ*M DAF-FM DA. After a further 30 min of incubations at 37°C, cells were rinsed twice with ice-cold PBS and harvested by trypsin. DAF-FM fluorescence intensity was measured via a FACScan flow cytometer (BECTON DICKINSON, USA) with excitation wave length at 495 nm and emission wave length at 515 nm for NO generation.

### 2.7. Western Blot Analysis

BV-2 cells treated with NaF were washed twice with PBS and then dissolved in 0.1 mL lysis buffer. Resulting lysates were centrifuged at 12,000 ×g for 3 min at 4°C in an ultracentrifuge. The protein concentrations of total cell lysates were measured using the Bio-Rad microprotein assay reagent and evenly adjusted. The protein extracts (30 *μ*g) were separated on SDS-PAGE and transferred to a polyvinylidene-difluoride (PVDF) membrane at 15 volt 40 min. The membranes were blocked in PBST blocking buffer (5% BSA in PBS with 0.05% Tween 20, pH 7.4) for 2 h. This was followed by incubating with primary antibodies overnight at 4°C. After three washes with PBST, the blots were incubated with appropriate horseradish peroxidase-linked secondary antibodies for 1 h at room temperature. The blots were washed again and detected the proteins of interest by Western Blotting Luminol Reagent according to the manufacturer's instructions, and then the chemiluminescence signal was visualized with X-ray film. Bands of NCAM isoforms were obtained and densitometric analysis was performed with Gel-Pro Analyzer version 3.0 software.

### 2.8. Enzyme Linked Immunosorbent Assays (ELISA)

After BV-2 cells were treated with NaF for 24 h, TNF-*α* and IL-1*β* in the cell culture media were measured using an ELISA kit according to the instructions of the manufacturer. The concentrations of TNF-*α* and IL-1*β* were calculated according to the standard curve of the ELISA kits.

### 2.9. Statistical Analysis

Statistical analyses were accomplished with SPSS for Windows, version 17.0. All experiments were performed at least in triplicate and the values represent mean ± SD. Differences between groups were statistically analyzed by one-way analysis of variance (ANOVA). Student's unpaired *t* test was used to compare two independent groups (antioxidant-treated versus antioxidant-untreated or inhibitor-treated versus inhibitor-untreated). *P* values <0.05 were designated as statistically significant.

## 3. Results

### 3.1. Effects of Fluoride on BV-2 Cell Viability

As shown in Table 1, BV-2 cell viability increased at 1 and 5 mg/L NaF-treated groups following 6 h of culture and then came back to the level of control group accompanying the increase of concentrations of NaF. After treatment with NaF for 12 h, the survival rates of 50 and 100 mg/L fluoride-treated groups were remarkably lower than that of the control group (*P* < 0.05). There were significant negative correlations between cell viability and NaF concentrations, and the correlation coefficients were −0.766, −0.809, −0.826 and −0.684 for 12 h, 24 h, 48 h, and 72 h, respectively. 

### 3.2. BV-2 Cell Activation after Being Treated with Fluoride

**Figure 1:**
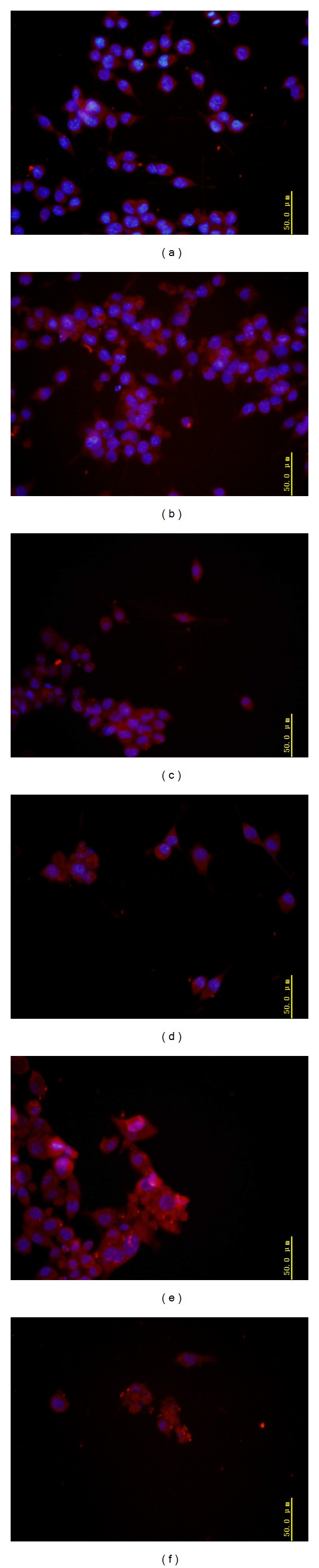
The activation of microglial BV-2 cells induced by fluoride. Cells were treated with indicated concentrations of NaF for 24 h and immunofluorescence localization with an Iba-1 antibody as a microglial marker being observed. Microglial activity was detected by Iba-1 expression (red) in cytoplasm and nucleus dyed with DAPI was presented in blue. Highly expression areas of Iba-1 immunoreactivity were indicated by red colour. Fluorescence microscopy: (400x). (a) Control, (b) 1 mg/L NaF, (c) 5 mg/L NaF, (d) 10 mg/L NaF, (e) 50 mg/L NaF, and (f) 100 mg/L NaF.

Iba-1 is as an ionized calcium-binding adaptor molecule. Expression level of Iba-1 increases as microglial cells were activated and immunocytochemistry localization of Iba-1 can be used as a microglia marker. Iba-1 expressions significantly increased in BV-2 microglia treated with various concentrations of NaF for 24 h and shown in bright red fluorescence in the cytoplasm in the dose-dependent manner, which indicated that fluoride can stimulate BV-2 cells to change into activated microglia displaying upregulated Iba-1 expression (Figure 1).

### 3.3. Effects of Fluoride on the Release of Inflammatory Cytokines

**Figure 2:**
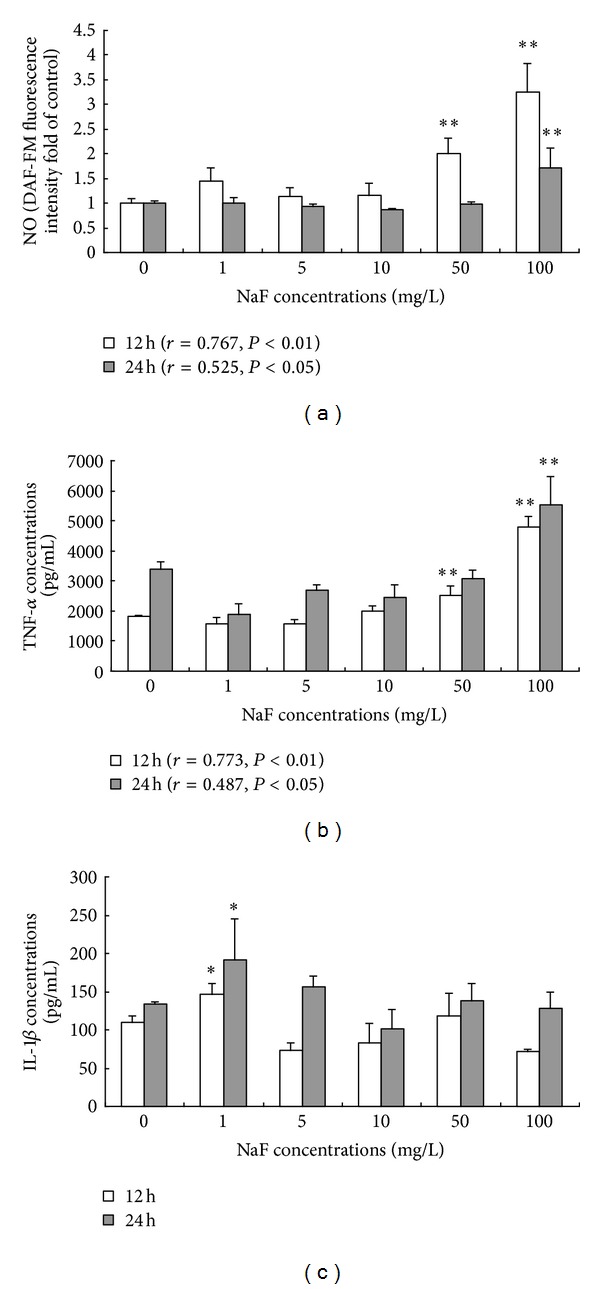
Effects of fluoride on the release of inflammatory cytokines of NO (a), TNF-*α* (b), and IL-1*β* (c) in BV-2 cells. Intracellular DAF-FM fluorescence intensity for NO and concentrations of TNF-*α* and IL-1*β* in the cell culture media were measured by flow cytometry and ELISA kits, respectively. Bars were presented as mean ± SD. *P < 0.05 and **P < 0.01 compared to the control group.

It was observed that the intracellular NO level increased significantly in fluoride-exposed cells in dose-dependent manner after being exposed NaF for 12 hours (*r* = 0.767, *P* < 0.01) and 24 hours (*r* = 0.525, *P* < 0.05). As shown in Figure 2(a), 50 and 100 *μ*g/mL NaF significantly induced NO release.  Figure 2(b) showed that BV-2 cells treated with 50 and 100 *μ*g/mL NaF exhibited significantly increased TNF-*α* release compared to control cells at exposure for 12 h (*P* < 0.01). The concentration of TNF-*α* in the cell culture media increased only in 100 *μ*g/mL NaF-treated BV-2 cells after being exposed NaF for 24 h. For the release of IL-1*β* in fluoride-treated BV-2 cells, we found that only 1 *μ*g/mL NaF induced significant increase of IL-1*β* compared to control group at exposure 12 h and 24 h (*P* < 0.05) see Figure 2(c). 

### 3.4. Effects of NOX and iNOS on the Oxidative Stress of BV-2 Cells Treated by Fluoride

**Figure 3:**
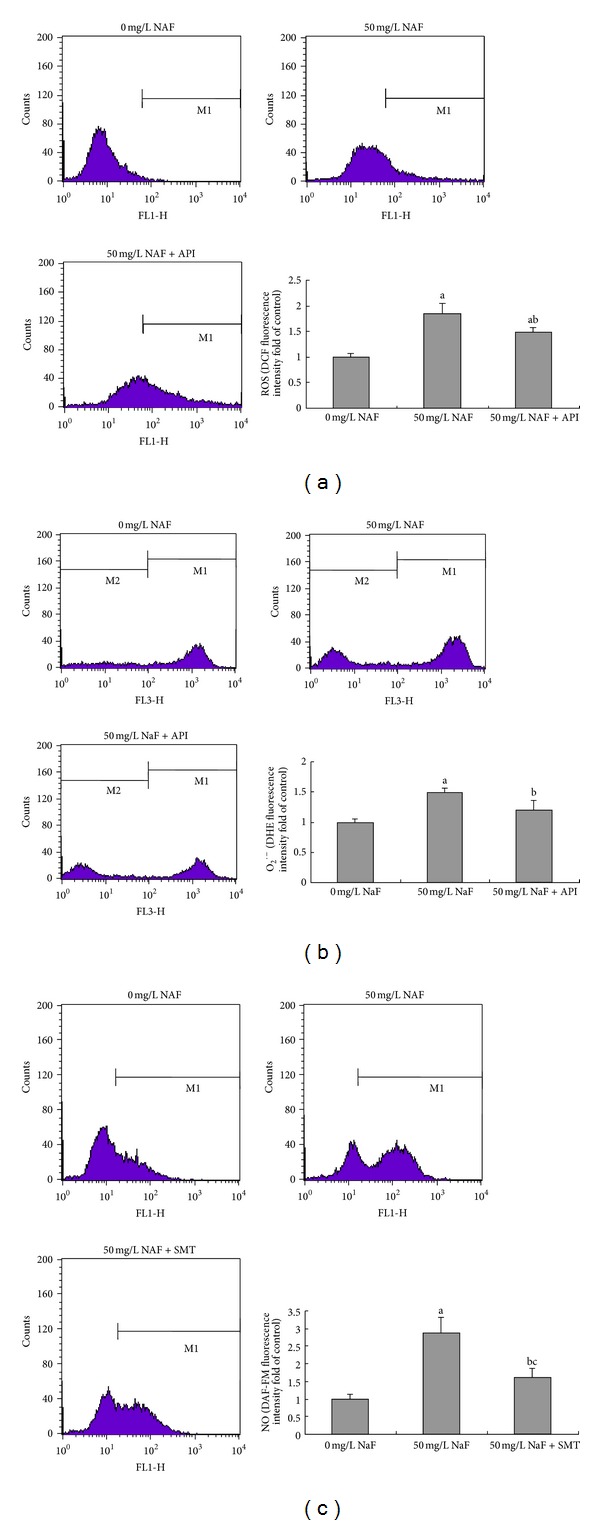
Effects of NADPH oxygenase and iNOS on the oxidative stress of BV-2 cells treated by fluoride. Intracellular DCF (a) and DHE (b) fluorescence intensity for ROS and O_2_^·−^ contents were measured by flow cytometer in BV-2 cells cotreated with 50 mg/L NaF and NADPH oxygenase inhibitor (API). Intracellular NO (c) was detected by measuring DAF-FM fluorescence in BV-2 cells cotreated with 50 mg/L NaF and iNOS inhibitor (SMT). Bars were presented as mean ± SD. a: *P* < 0.01 compared to 0 mg/L NaF group; b: *P* < 0.01 compared to 50 mg/L NaF group; c: *P* < 0.05 compared to 0 mg/L NaF group.

Our previous study had shown that fluoride could cause oxidative stress and induce the increase of intracellular ROS and O_2_^·−^ in BV-2 microglial cells [13]. NOX has been characterized as the main source of intracellular ROS during microglial activation and is activated in a variety of microglial activation processes. To determine whether NOX and iNOS were involved in fluoride-mediated oxidative stress in BV-2 cells, we tested the effects of the NOX and iNOS. Pretreatment of BV-2 cells with NOX inhibitor API dramatically decreased NaF-induced ROS and O_2_^·−^ generations. The contents of intracellular ROS and O_2_^·−^ were evaluated by the changes in DCF and DHE fluorescence intensity, respectively, and shown in Figures 3(a) and 3(b). In fluoride-treated BV-2 cells, pretreatment with iNOS inhibitor SMT also effectively reduced intracellular NO level compared with the cells with only fluoride treatment. This further confirms that NOX and iNOS play an important role in fluoride inducing oxidative stress and NO production (Figure 3(c)).

### 3.5. Effects of MAPK Signal Pathway on Fluoride-Induced Oxidative Stress in BV-2 Cells

**Figure 4:**
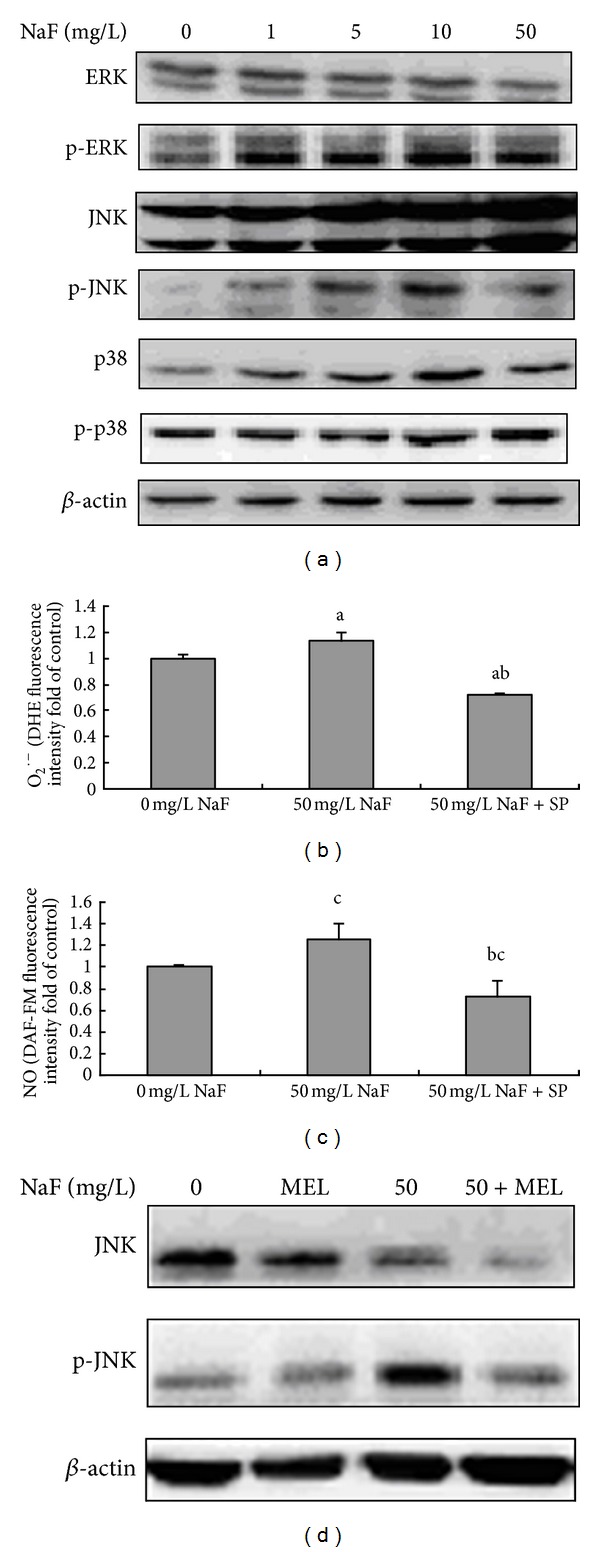
JNK/MAPK signal pathway is involved in fluoride-induced oxidative stress of BV-2 cells. (a) BV-2 cells were treated with 1, 5, 10, and 50 mg/L NaF for 24 h, and cell lysates were separated on 10–20% SDS-PAGE and analyzed by Western blot. (b) and (c) Intracellular DHE and DAF-FM fluorescence intensity for O_2_^·−^ and NO contents were measured by flow cytometer in BV-2 cells preincubated with 50 *μ*M JNK/MAPK inhibitor (SP600125) for 2 h, followed by 50 mg/L NaF for 24 h. (d) BV-2 cells were pretreated with 2 mM MEL for 2 h, followed by 50 mg/L NaF for 24 h, and cell lysates were blotted with antiphospho (p)-JNK or anti-JNK antibodies. Bars were presented as mean ± SD. a: *P* < 0.01 compared to 0 mg/L NaF group; b: *P* < 0.01 compared to 50 mg/L NaF group; c: *P* < 0.05 compared to 0 mg/L NaF group.

It has been previously shown that NaF increases ERK, p38, and JNK activity in human lung epithelial cells [23, 24]. We examined here the expressions of MAPK in NaF-treated BV-2 microglia cells. Western blot showed that treatment with 5, 10, and 50 *μ*g/mL NaF increased JNK phosphorylation level but did not influence ERK and p38 phosphorylation (Figure 4(a)). To further understand that the possible effects of MAPK signal pathway in fluoride induce oxidative stress, BV-2 cells were treated with 50 *μ*g/mL NaF in the presence or absence of JNK inhibitor SP600125. As shown in Figures 4(b) and 4(c), JNK inhibitor SP600125 markedly reduced the levels of intracellular O_2_^·−^ and NO. Our study herein suggested that fluoride could cause JNK phosphorylation, which also took part in the oxidative stress induced by fluoride in BV-2 microglial cells. Several lines suggest that ROS production follows the activation of p38 MAPK [25]; the effect of ROS on the activation of JNK was investigated by the means of Western blot analysis. We found that treatment with antioxidant MEL resulted in a reduction in JNK phosphorylation in fluoride-stimulated BV-2 microglia (Figure 4(d)), which means that JNK activation requires ROS in fluoride-treated microglia. Together, our data provided the evidence that JNK took part in the oxidative stress induced by fluoride and meanwhile also could be activated by ROS in fluoride-treated BV-2 cells. 

## 4. Discussion

Fluorine is an essential trace element for the body, but excessive fluoride intake over a long period of time may result in dental and skeletal fluorosis, as well as the deterioration of the learning and memory capability and reduction of IQ [26, 27]. Fluoride exposure increased the generation of ROS [28, 29], which seems particularly important in mediating fluoride's effects. Oxidative stress is a recognized mode of action of fluoride exposure that has been observed in vitro in several types of cells and also in vivo in soft tissues, and oxidative damage is the major mode of action of fluoride.

Microglia are the resident macrophages of the brain and as such play critical roles in the development and maintenance of the neural environment [30]. Although microglia continually survey the surrounding tissue, they remain in essentially a quiescent state under tight regulation until they become activated in response to perturbations in the brain's microenvironment or changes in the neuronal structure. Iba-1 is a marker of microglia activation, and here we observed that intracellular Iba-1 expression level was heightened in fluoride exposure groups, which means that the activation of microglia is induced by fluoride. Once activated, microglial cells produce a wide variety of inflammatory mediators which serve to mediate an innate immune response, including TNF-*α*, IL-1*β*, NO, and ROS. TNF-*α* and IL-1*β* are the two main proinflammatory cytokines produced by activated microglia during inflammation caused by the disruption of the brain-blood barrier (BBB) [31, 32]. Interestingly, our work showed that IL-1*β* level is significantly enhanced only in 1 mg/L NaF-treated BV-2cells and retrieved with fluoride concentrations being increased. NO and TNF-*α* levels increased significantly in 50 and 100 mg/L NaF-treated groups at 12 h and only 100 mg/L NaF-treated groups at 24 h compared with control group, although in a dose-dependent manner with fluoride exposure concentrations. These observations indicated that fluoride may promote IL-1*β* secretion in low dose and NO and TNF-*α* secretion in high dose. These results also proved further that fluoride could stimulate microbial cells activation. 

Our previous research has revealed that fluoride can attack oxygen species, and additional ROS are generated. Several intracellular sources contribute to ROS generation in monocytes, including cyclooxygenases, lipoxygenases, mitochondrial respiration, and NOX [33]. NOX is the major enzyme for the production of extracellular superoxide in immune cells and is highly expressed in microglia. We have found that API, a widely used NOX inhibitor, can reduce the O_2_^·−^ and ROS level significantly. It has also been demonstrated that the iNOS inhibitor, SMT, can inhibit the production of NO. These observations suggest that the oxidative stress response is more specifically mediated by NOX and iNOS. 

MAPKs are important kinases in microglial redox signaling [34, 35] and control some gene expression of proinflammatory cytokines, chemokines, and enzymes [36, 37]. JNK and p38 are known as stress-activated protein kinases and play key roles in cellular stress, apoptosis, and inflammation [38, 39]. Here, we observed that p-JNK expression is enhanced with exposure to sodium fluoride in BV-2 microglial cells, which were consistent with other reports that fluoride affected MAPK signaling pathways in some cell types [22, 40]. The JNK pathway can be activated by a variety of cellular stressors, including TNF-*α* [41]. TNF-*α* induced by fluoride might mediate JNK phosphorylation in BV-2 microglia. 

ROS can act as an important mediator to activate various signaling molecules and pathways, including the MAPK pathway [42, 43], while phosphorylated MAPKs also could produce more ROS. In the present study, we found that antioxidant MEL, participating in diverse physiological functions, significantly inhibited p-JNK expression. MEL has been demonstrated the neuroprotective effects in various experiment, based on its antioxidant activity [44, 45]. Our results suggested that ROS might play part role in the activation of JNK-signaling pathway. To further analyze the role of JNK pathways in the oxidative stress induced by fluoride, JNK/MAPK signaling was blocked by inhibitors for JNK, SP600125. The results showed that O_2_^·−^ and NO levels decreased by inhibition of JNK by SP600125 in fluoride-treated BV-2 cells. These findings indicated that JNK signaling is responsible for fluoride-induced ROS and NO increase. These results suggest that oxygen radicals can work both upstream and downstream of the JNK activation in fluoride-treated BV-2 microglia.

In conclusion, we have identified that fluoride stimulated BV-2 microglial cells activation and consequently resulted in inflammatory cytokines releasing and oxidative stress strengthen. ROS generations induced by fluoride were part of activation of JNK/MAPK signaling pathway and NOX, while ROS were also as upstream of JNK/MAPK signaling pathway to activate JNK. Our results suggest that inflammatory cytokines and ROS releasing from activated microglial cells induced by fluoride might exert neurotoxicity in the toxic effects of fluoride on the central nervous system. 

## Figures and Tables

**Table 1:** Effects of fluoride on cell viability in microglial BV-2 cells (the percentage against the control group, %).

NaF (*μ*g/mL)	6 h	12 h	24 h	48 h	72 h
0	100.0 ± 3.6	100.0 ± 1.7	100.0 ± 0.0	100.0 ± 5.1	100.0 ± 0.9
1	107.7 ± 4.6**	100.0 ± 2.1	95.0 ± 1.0**	90.2 ± 3.7**	102.2 ± 3.4
5	106.8 ± 2.2*	98.4 ± 3.5	96.0 ± 1.0**	93.2 ± 3.3*	100.2 ± 3.1
10	102.6 ± 4.5	94.5 ± 1.4*	94.3 ± 1.6*	92.3 ± 1.9*	102.2 ± 3.1
25	101.4 ± 5.6	94.8 ± 5.9**	91.6 ± 5.4**	84.4 ± 5.8**	102.7 ± 2.2
50	100.2 ± 5.1	93.9 ± 2.6*	87.1 ± 4.2**	81.0 ± 5.0**	86.4 ± 1.6**
100	90.3 ± 1.8**	78.1 ± 1.4**	70.7 ± 6.2*	71.8 ± 7.7**	50.2 ± 2.9**

*R*	−0.517	−0.766	−0.809	−0.826	−0.684
*P*	0.002	0.000	0.000	0.000	0.000

*R*: the correlation coefficients; *P*: *P* values; *P < 0.05 compared with 0 *μ*g/mL group; **P < 0.01 compared with 0 *μ*g/mL group.

## References

[1] Ozsvath DL (2009). Fluoride and environmental health: a review. *Reviews in Environmental Science and Biotechnology*.

[2] Ekambaram P, Paul V (2001). Calcium preventing locomotor behavioral and dental toxicities of fluoride by decreasing serum fluoride level in rats. *Environmental Toxicology and Pharmacology*.

[3] Paul V, Ekambaram P, Jayakumar AR (1998). Effects of sodium fluoride on locomotor behavior and a few biochemical parameters in rats. *Environmental Toxicology and Pharmacology*.

[4] Bhatnagar M, Rao P, Jain S, Bhatnagar R (2002). Neurotoxicity of fluoride: neurodegeneration in hippocampus of female mice. *Indian Journal of Experimental Biology*.

[5] Choi AL, Sun G, Zhang Y, Grandjean P (2012). Developmental fluoride neurotoxicity: a systematic review and meta-analysis. *Environmental Health Perspectives*.

[6] Xiang Q, Liang Y, Chen L (2003). Effect of fluoride in drinking water on children’s intelligence. *Fluoride*.

[7] Tang QQ, Du J, Ma HH, Jiang SJ, Zhou XJ (2008). Fluoride and children’s intelligence: a meta-analysis. *Biological Trace Element Research*.

[8] Zhang M, Wang A, He W (2007). Effects of fluoride on the expression of NCAM, oxidative stress, and apoptosis in primary cultured hippocampal neurons. *Toxicology*.

[9] Butterfield DA (2006). Oxidative stress in neurodegenerative disorders. *Antioxidants and Redox Signaling*.

[10] Donnelly DF, Carroll JL (2005). Mitochondrial function and carotid body transduction. *High Altitude Medicine & Biology*.

[11] Chinoy NJ, Patel TN (2000). The influence of fluoride and/or aluminium on free radical toxicity in the brain of female mice and beneficial effects of some anti-dotes. *Fluoride*.

[12] Georgieva NV (2005). Oxidative stress as a factor of disrupted ecological oxidative balance in biological systems—a review. *Bulgarian Journal of Veterinary Medicine*.

[13] Shuhua X, Ziyou L, Ling Y, Fei W, Guifan S (2012). A role of fluoride on free radical generation and oxidative stress in BV-2 microGlia cells. *Mediators of Inflammation*.

[14] Aloisi F (2001). Immune function of microGlia. *Glia*.

[15] Rock RB, Gekker G, Hu S (2004). Role of microGlia in central nervous system infections. *Clinical Microbiology Reviews*.

[16] McGeer PL, Itagaki S, Boyes BE, McGeer EG (1988). Reactive microGlia are positive for HLA-DR in the substantia nigra of Parkinson’s and Alzheimer’s disease brains. *Neurology*.

[17] Giulian D, Haverkamp LJ, Li J (1995). Senile plaques stimulate microGlia to release a neurotoxin found in Alzheimer brain. *Neurochemistry International*.

[18] Rockwell P, Martinez J, Papa L, Gomes E (2004). Redox regulates COX-2 upregulation and cell death in the neuronal response to cadmium. *Cellular Signalling*.

[19] Svensson C, Fernaeus SZ, Part K, Reis K, Land T (2010). LPS-induced iNOS expression in Bv-2 cells is suppressed by an oxidative mechanism acting on the JNK pathway—a potential role for neuroprotection. *Brain Research*.

[20] Pocivavsek A, Burns MP, Rebeck GW (2009). Low-density lipoprotein receptors regulate microGlial inflammation through c-Jun N-terminal kinase. *Glia*.

[21] Emre Y, Hurtaud C, Nübel T, Criscuolo F, Ricquier D, Cassard-Doulcier A-M (2007). Mitochondria contribute to LPS-induced MAPK activation via uncoupling protein UCP2 in macrophages. *Biochemical Journal*.

[22] Thrane EV, Refsnes M, Thoresen GH, Lag M, Schwarze PE (2001). Fluoride-induced apoptosis in epithelial lung cells involves activation of MAP kinases p38 and possibly JNK. *Toxicological Sciences*.

[23] Refsnes M, Skuland T, Schwarze PE, Ovrevik J, Lag M (2008). Fluoride-induced IL-8 release in human epithelial lung cells: relationship to EGF-receptor-, SRC- and MAP-kinase activation. *Toxicology and Applied Pharmacology*.

[24] Refsnes M, Thrane EV, Lag M, Hege Thoresen G, Schwarze PE (2001). Mechanisms in fluoride-induced interleukin-8 synthesis in human lung epithelial cells. *Toxicology*.

[25] Yuan H, Perry CN, Huang C (2009). LPS-induced autophagy is mediated by oxidative signaling in cardiomyocytes and is associated with cytoprotection. *American Journal of Physiology*.

[26] Bruce S (2000). Fluoride and intelligence. *Fluoride*.

[27] Chioca LR, Raupp IM, Da Cunha C, Losso EM, Andreatini R (2008). Subchronic fluoride intake induces impairment in habituation and active avoidance tasks in rats. *European Journal of Pharmacology*.

[28] García-Montalvo EA, Reyes-Pérez H, Del Razo LM (2009). Fluoride exposure impairs glucose tolerance via decreased insulin expression and oxidative stress. *Toxicology*.

[29] Izquierdo-Vega JA, Sánchez-Gutiérrez M, Del Razo LM (2008). Decreased in vitro fertility in male rats exposed to fluoride-induced oxidative stress damage and mitochondrial transmembrane potential loss. *Toxicology and Applied Pharmacology*.

[30] Kraft AD, Jean Harry G (2011). Features of microGlia and neuroinflammation relevant to environmental exposure and neurotoxicity. *International Journal of Environmental Research and Public Health*.

[31] Chao CC, Hu S, Ehrlich L, Peterson PK (1995). Interleukin-1 and tumor necrosis factor-*α* synergistically mediate neurotoxicity: involvement of nitric oxide and of *N*-methyl-D-aspartate receptors. *Brain, Behavior, and Immunity*.

[32] Chew L-J, Takanohashi A, Bell M (2006). MicroGlia and inflammation: impact on developmental brain injuries. *Mental Retardation and Developmental Disabilities Research Reviews*.

[33] Irani K (2000). Oxidant signaling in vascular cell growth, death, and survival: a review of the roles of reactive oxygen species in smooth muscle and endothelial cell mitogenic and apoptotic signaling. *Circulation Research*.

[34] Bhat NR, Zhang P, Lee JC, Hogan EL (1998). Extracellular signal-regulated kinase and p38 subgroups of mitogen- activated protein kinases regulate inducible nitric oxide synthase and tumor necrosis factor-*α* gene expression in endotoxin-stimulated primary Glial cultures. *Journal of Neuroscience*.

[35] Hidding U, Mielke K, Waetzig V (2002). The c-Jun N-terminal kinases in cerebral microGlia: immunological functions in the brain. *Biochemical Pharmacology*.

[36] Baldwin AS (2001). The transcription factor NF-*κ*B and human disease. *Journal of Clinical Investigation*.

[37] Chandrasekar B, Freeman GL (1997). Induction of nuclear factor *κ*B and activation protein 1 in postischemic myocardium. *FEBS Letters*.

[38] Chen Z, Gibson TB, Robinson F (2001). MAP kinases. *Chemical Reviews*.

[39] Kyriakis JM, Avruch J (1996). Sounding the alarm: protein kinase cascades activated by stress and inflammation. *Journal of Biological Chemistry*.

[40] Lau KH, Baylink DJ (1998). Molecular mechanism of action of fluoride on bone cells. *Journal of Bone and Mineral Research*.

[41] Karmann K, Min W, Fanslow WC, Pober JS (1996). Activation and homologous desensitization of human endothelial cells by CD40 ligand, tumor necrosis factor, and interleukin 1. *Journal of Experimental Medicine*.

[42] McCubrey JA, LaHair MM, Franklin RA (2006). Reactive oxygen species-induced activation of the MAP kinase signaling pathways. *Antioxidants & Redox Signaling*.

[43] Torres M, Forman HJ (2003). Redox signaling and the MAP kinase pathways. *Biofactors*.

[44] Reiter RJ, Melchiorri D, Sewerynek E (1995). A review of the evidence supporting melatonin’s role as an antioxidant. *Journal of Pineal Research*.

[45] Agrawal R, Tyagi E, Shukla R, Nath C (2008). Effect of insulin and melatonin on acetylcholinesterase activity in the brain of amnesic mice. *Behavioural Brain Research*.

